# Compressive-Shear Behavior and Cracking Characteristics of Composite Pavement Asphalt Layers Under Thermo-Mechanical Coupling

**DOI:** 10.3390/ma18194543

**Published:** 2025-09-30

**Authors:** Shiqing Yu, You Huang, Zhaohui Liu, Yuwei Long

**Affiliations:** 1 School of Transportation, Changsha University of Science and Technology, Changsha 410114, China; 22001090016@csust.edu.cn (S.Y.); liuzhaohui@csust.edu.cn (Z.L.); 2 Engineering Research Center of Catastrophic Prophylaxis and Treatment of Road & Traffic Safety of Ministry of Education, Changsha University of Science & Technology, Changsha 410114, China; 3 Hunan Expressway Maintenance Engineering Co., Ltd., Changsha 410003, China; longyw9@163.com

**Keywords:** road engineering, compression-shear stress, thermo-mechanical coupling, composite pavement, top-down cracking

## Abstract

Cracking in asphalt layers of rigid–flexible composite pavements under coupled ambient temperature fields and traffic loading represents a critical failure mode. Traditional models based on uniform temperature assumptions inadequately capture the crack propagation mechanisms. This study developed a thermo-mechanical coupling model that incorporates realistic temperature-modulus gradients to analyze the compressive-shear behavior and simulate crack propagation using the extended finite element method (XFEM) coupled with a modified Paris’ law. Key findings reveal that the asphalt layer exhibits a predominant compressive-shear stress state; increasing the base modulus from 10,000 MPa to 30,000 MPa reduces the maximum shear stress by 22.8% at the tire centerline and 8.6% at the edge; thermal stress predominantly drives crack initiation, whereas vehicle loading governs the propagation path; field validation via cored samples confirms inclined top-down cracking under thermo-mechanical coupling; and the fracture energy release rate (*G_f_*) reaches a minimum of 155 J·m^−2^ at 14:00, corresponding to a maximum fatigue life of 32,625 cycles, and peaks at 350 J·m^−2^ at 01:00, resulting in a reduced life of 29,933 cycles—reflecting a 9.0% temperature-induced fatigue life variation. The proposed model, which integrates non-uniform temperature gradients, offers enhanced accuracy in capturing complex boundary conditions and stress states, providing a more reliable tool for durability design and assessment of composite pavements.

## 1. Introduction

Rigid–flexible composite pavement, as a high-performance pavement structure, has been widely adopted due to its exceptional load-bearing capacity and driving comfort [1,2,3]. However, its asphalt layer is typically relatively thin and is particularly sensitive to variations in environmental temperature [4,5]. Extensive field measurements and studies have indicated that complex service environments, especially temperature fields, can lead to significant spatial variability in the material parameters of the asphalt layer in composite pavements [6,7]. This is manifested as a nonlinear temperature distribution along the depth and a consequent dynamic gradient in modulus distribution [8,9,10]. Such spatial heterogeneity in material properties can fundamentally alter the mechanical response of the pavement structure, resulting in increased tensile stress at the road surface and elevated maximum shear stress, thereby exacerbating the risk of top-down cracking in asphalt pavements [11,12,13,14]. Observations from the RIOHTrack corroborate this phenomenon, where top-down transverse cracks in the wheel path, differing from traditional fatigue damage patterns, have emerged as a primary failure mode for composite pavement structure [15,16].

Regarding the simulation of fracture behavior in asphalt pavements, numerous studies have been conducted by scholars worldwide. In terms of analytical methods, DONG et al. [11] established a layered system theoretical model that accounts for the modulus gradient, elucidating the mechanical mechanism of top-down cracking. Within the realm of numerical simulation, the finite element method (FEM) is a mainstream tool. For instance, LING et al. [6] investigated the S-shaped influence pattern of temperature on the response of airport composite pavements, while TAO et al. [17] and XIE et al. [13] studied the effects of interlayer bonding conditions and the propagation of reflective cracks, respectively. However, most of these studies are based on traditional FEM, which faces significant computational challenges associated with mesh remeshing when dealing with discontinuous crack problems.

To overcome this limitation, advanced numerical methods based on fracture mechanics have been introduced into the field of pavement engineering [16,18,19]. Among these, the XFEM allows cracks to grow independently within elements without the need for mesh reconstruction, and it has been successfully applied to simulate reflective cracking and top-down cracking [20,21,22]. On the other hand, discrete fracture methods, such as the cohesive zone model, have increasingly been used in recent years for simulating cracks in quasi-brittle materials and pavement materials [23]. This method naturally describes crack initiation and propagation by embedding interface elements with a traction–separation constitutive law along potential fracture paths. Umberto et al. [24] proposed a numerical strategy based on the cohesive zone model for simulating fracture in heterogeneous materials, ensuring accurate representation of complex fracture mechanisms while maintaining high computational efficiency. LIU et al. [25] applied this model to effectively predict crack propagation behavior and the remaining service life of asphalt pavements under different coupled conditions of temperature and load. Furthermore, Mohsen et al. [26] introduced fracture mechanics into a linear elastic model to study the dynamic response of porous asphalt pavements. These studies demonstrate the significant potential of discrete fracture methods in addressing complex environmental–mechanical coupled fracture problems.

Despite the progress mentioned above, the existing research still has notable limitations. Firstly, most studies have focused on the influence of single factors such as load, material properties, or structural layer combinations, with relatively insufficient research on the thermo-mechanical coupling under the combined action of dynamic temperature fields and moving loads in actual service environments [27,28,29,30]. Secondly, although Darabi et al. [31] coupled aging and damage, their model was a simplified 2D model, which has discrepancies with real 3D pavement structures in terms of boundary conditions and stress states, making it difficult to accurately capture three-dimensional crack propagation behavior. Although both XFEM and the cohesive zone model have their applications, there is a lack of comparative research on these two methods for simulating crack propagation in rigid–flexible composite pavements under temperature gradients, particularly concerning the analysis of the “compressive-shear” composite stress characteristics within the asphalt layer.

**Figure 1:**
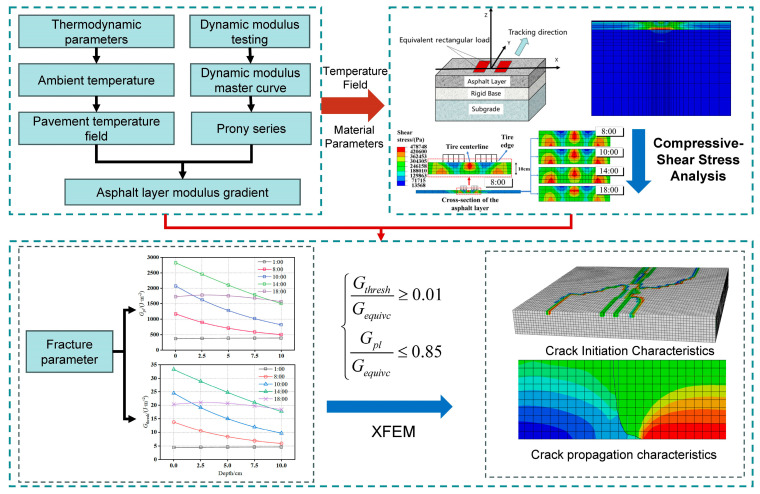
Methodological framework of this paper.

Addressing these research gaps, the innovative aspects of this study are as follows: establishing a 3D thermo-mechanical coupled finite element model of rigid–flexible composite pavement validated with field measurement data to systematically analyze the dynamic modulus distribution of the asphalt layer under actual temperature gradients and its impact on the mechanical response; focusing on the compressive-shear composite stress characteristics of the asphalt layer under thermo-mechanical coupling; and employing the XFEM to simulate and analyze the cracking behavior of the asphalt layer in rigid–flexible composite pavements considering actual temperature fields, thereby revealing its crack propagation characteristics. The methodological framework of this paper is illustrated in Figure 1.

## 2. Theory and Methodology

### 2.1. Boundary Condition of Pavement Temperature Field

The diurnal variation of solar radiation was approximated using the following piecewise function shown in Equation (1) [32]:

(1) $$q\left(t\right)=\left\{\begin{array}{ll} 0 & 0\leq t<12-\frac{c}{2}\\ q_{0}\cos m\omega (t-12) & 12-\frac{c}{2}\leq t\leq 12+\frac{c}{2}\\ 0 & 12+\frac{c}{2}<t\leq 24 \end{array}\right.$$

where *q*_0_ is the maximum solar radiation at noon, given by *q*_0_ = 0.131*Qm* with *m* = 12/*c*; *Q* is the total daily solar radiation (J·m^−2^); *c* is the actual effective sunshine duration (hours); and *ω* is the angular frequency (rad).

Since Equation (1) was not a continuous function, it was expanded into a Fourier series, as shown in Equation (2).

(2) $$q(t)=\frac{a_{0}}{2}+\sum_{k=1}^{\infty }a_{k}\cos\frac{k\pi \left(t-12\right)}{12}$$

where $a_{0}=\frac{2q_{0}}{m\pi }$.

The Fourier coefficients *a_k_* are given by Equation (3):

(3) $$a_{k}=\left\{\begin{array}{ll} \frac{q_{0}}{\pi }\left[\frac{1}{m+k}\sin\left(m+k\right)\frac{\pi }{2m}+\frac{\pi }{2m}\right] & k=m\\ \frac{q_{0}}{\pi }\left[\frac{1}{m+k}\sin\left(m+k\right)\frac{\pi }{2m}+\frac{1}{m-k}\sin\left(m-k\right)\frac{\pi }{2m}\right] & k\neq m \end{array}\right.$$

Solar radiation was simulated in ABAQUS 2020 finite element software using the Load module in combination with a user-defined DFLUX subroutine, implementing the radiation model described by Equations (1)–(3). The diurnal variation of air temperature was modeled using Equation (4), which represents a linear combination of two sinusoidal functions.

(4) $$T_{a}=\bar{T_{a}}+T_{m}\left[0.96\sin\omega \left(t-t_{0}\right)+0.14\sin2\omega \left(t-t_{0}\right)\right]$$

where $\bar{T_{a}}$ is the mean daily air temperature (°C); $T_{m}$ is the daily air temperature amplitude (°C), given by $T_{m}=\frac{1}{2}\left(T_{a}^{\max}-T_{a}^{\min}\right)$, where $T_{a}^{\max}$ and $T_{a}^{\min}$ are the daily maximum and minimum air temperatures, respectively; and $t_{0}$ is the initial phase shift (hours).

Air temperature and convective heat exchange were implemented in ABAQUS using the Interaction module along with the FILM subroutine, incorporating the temperature model from Equation (4).

The boundary condition for effective pavement radiation is expressed by Equation (5):

(5) $$q_{F}=\varepsilon \sigma \left[{\left(T_{1}{|}_{Z=0}-T_{Z}\right)}^{4}-{\left(T_{a}-T_{z}\right)}^{4}\right]$$

where $q_{F}$ is the effective surface radiation $W/\left(m^{2}\cdot {}^{\circ}C\right)$; ε is the pavement emissivity (blackness), taken as 0.81 for asphalt pavement; σ is the Stefan–Boltzmann constant, $\sigma =5.6697\times 10^{-8}$; $T_{1}{|}_{Z=0}$ is the pavement surface temperature (°C); $T_{a}$ is the air temperature (°C); and $T_{z}$ is the absolute zero value (°C), $T_{z}=-273\;{}^{\circ}C$.

**Figure 2:**
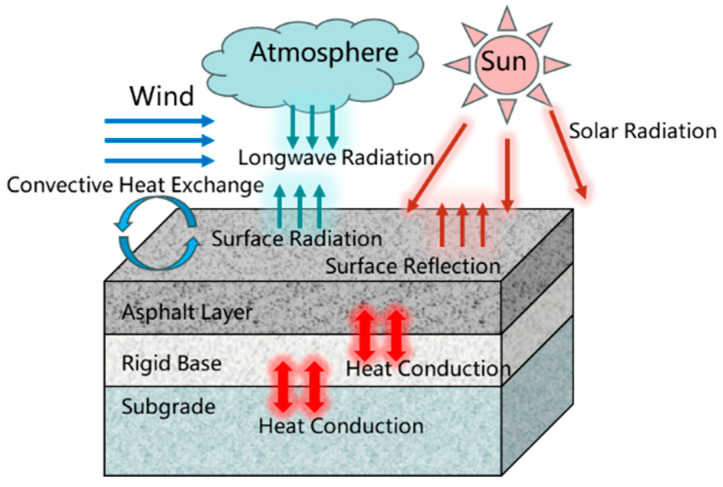
Schematic diagram of heat exchange modes in pavement.

Effective pavement radiation was defined within the Interaction module of ABAQUS using Equation (5). The modes of heat exchange between the pavement structure and the external environment are illustrated in Figure 2, which incorporates the mathematical formulations presented in Equations (1)–(5).

### 2.2. Modified Paris’ Law

**Figure 3:**
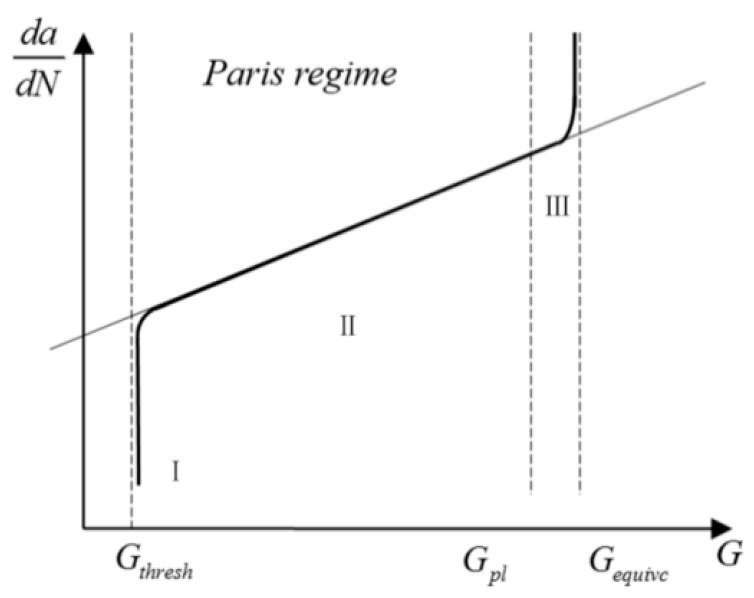
Modified Paris’ law curve [35,38].

This study develops an integrated computational framework combining the XFEM with a modified Paris’ law to predict cracking propagation path and fatigue growth rates. The XFEM resolves geometrical complexity in cracking propagation, while the modified Paris’ law quantifies propagation kinetics through physical mechanisms [33]. In fracture mechanics, the energy release rate (*G*) and stress intensity factor (*K*) constitute fundamental parameters characterizing cracking driving forces [34]. Substitution of *K* with *G* in the conventional Paris’ law formulation yields the modified version (Equation (6), Figure 3), which describes fatigue cracking progression through distinct propagation phases [35,36].

(6) $$\frac{da}{dN}=C^{\prime }{\left(\Delta G\right)}^{m^{\prime }}$$

where $C^{\prime }$ and $m^{\prime }$ are material parameters in the modified Paris’ law, with values of 3.5574 and 0.4606 for asphalt mixtures [37]. Δ*G* represents the difference in fracture energy release rate between the maximum and minimum alternating stresses, i.e., Δ*G* = *G*_max_ − *G*_min_. *da* denotes the cracking growth length, and *dN* represents the number of load cycles.

*G_thresh_* represents the threshold value of the fracture energy release rate. Cracking propagation only begins when the fracture energy release rate at the crack tip exceeds *G_thresh_*. *G_pl_* is the upper limit of the fracture energy release rate, indicating that when the fracture energy release rate exceeds *G_pl_*, fatigue cracking propagation enters Stage III, as shown in Figure 3. *G_equivc_* represents the critical energy release rate, also known as the material’s fracture toughness. For cracking propagation to occur, the relationship shown in Equation (7) must be satisfied.

(7) $$\left\{\begin{array}{c} \frac{G_{thresh}}{G_{equivc}}\geq 0.01\\ \frac{G_{pl}}{G_{equivc}}\leq 0.85 \end{array}\right.$$

Cracking propagation can be divided into three stages:

Stage I: When Δ*G* < *G_thresh_*, no cracking propagation occurs.

Stage II: When *G_thresh_* < Δ*G* < *G_equivc_*, the cracking is in a stable propagation phase. In this stage, the modified Paris’ law shown in Equation (6) is generally considered applicable for simulating cracking propagation in asphalt mixtures.

Stage III: When Δ*G* < *G_pl_*, the cracking enters an unstable propagation phase. In this stage, cracking grows rapidly, and fatigue life can be considered negligible.

## 3. FE Modeling and Validation

### 3.1. Pavement Structure Parameters

**Figure 4:**
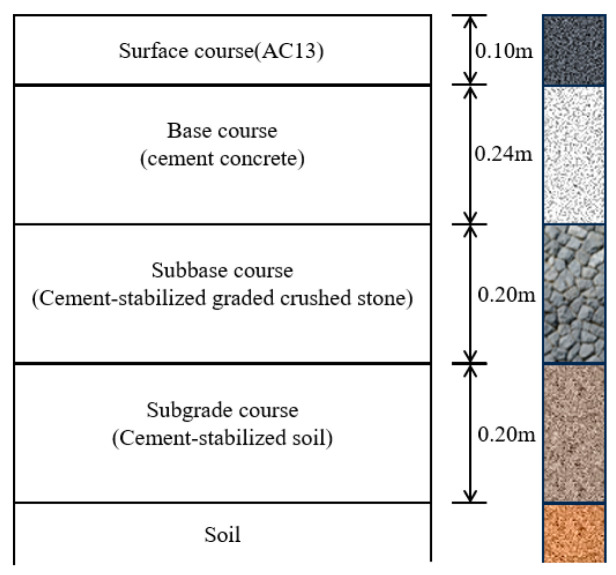
The simplified composite pavement structure.

**Figure 5:**
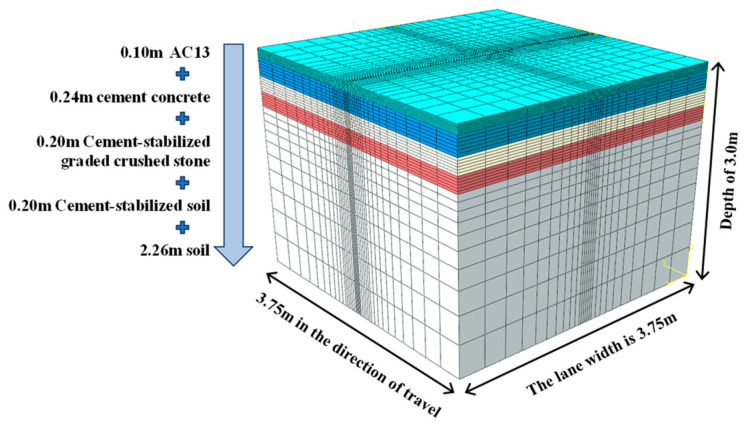
FE model and meshing.

The study employed ABAQUS software to develop a finite element model of the rigid–flexible composite pavement. The model dimensions were established as follows: 3.75 m in the driving direction, 3.75 m perpendicular to the driving direction (representing the standard lane width), and 3.0 m in the vertical depth. The material type and thickness of each layer for the simplified composite pavement structure are shown in Figure 4. The FE model of the pavement structure after meshing is shown in Figure 5.

### 3.2. Ambient Temperature

Meteorological data from Beijing’s RIOHTrack facility identified 21 July 2018 and 11 December 2018 as representative summer and winter days, respectively. Temperature measurements are summarized in Table 1, with external meteorological parameters detailed in Table 2. Table 3 presents the thermophysical properties of the investigated materials.

### 3.3. Material Properties

#### 3.3.1. Dynamic Modulus

**Figure 6:**
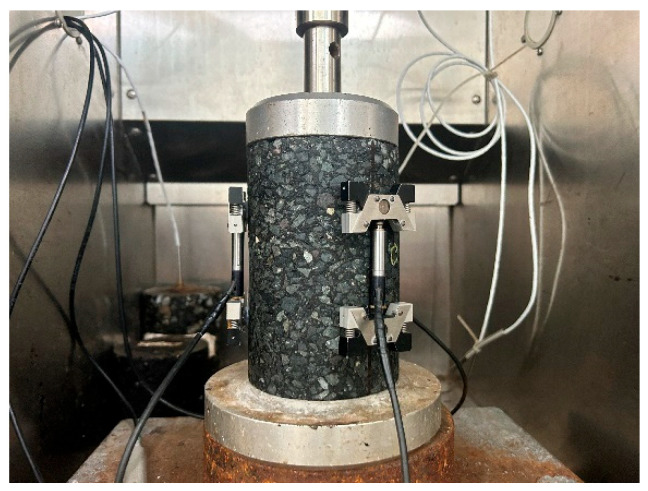
SPT testing.

Dynamic modulus testing was conducted using the Simple Performance Tester (SPT) following AASHTO TP62-03 [40] specifications [30]. Cylindrical specimens (∅100 × 150 mm) were prepared through a standardized fabrication protocol comprising rotary compaction, coring, dimensional calibration, and 6 h thermal equilibration in an environmental chamber (±0.5 °C accuracy). Preconditioning cycles were implemented to minimize experimental artifacts [41,42]. Each group of experiments was repeated three times. Figure 6 schematizes the SPT loading protocol, while Table 4 and Table 5, respectively, detail the experimental matrix and corresponding modulus values across the tested temperature–frequency domains.

The dynamic modulus master curve was modeled through nonlinear regression analysis employing the Boltzmann function (Equation (8)) with temperature as the predictor variable [43,44].

(8) $$\mathrm{lg}|E^{*}|=\frac{\mathrm{lg}|E_{\max}^{*}|-\mathrm{lg}|E_{\min}^{*}|}{1+e^{(T-T_{E0})/\mathrm{dx}}}+\mathrm{lg}|E_{\min}^{*}|$$

where lg|E^*^_min_| and lg|E^*^_max_|—Logarithmic bounds of dynamic modulus; *T*—Test temperature (°C); *T_E_*_0_—Characteristic temperature at peak slope of modulus-temperature curve (°C); and dx—Regression parameter.

**Figure 7:**
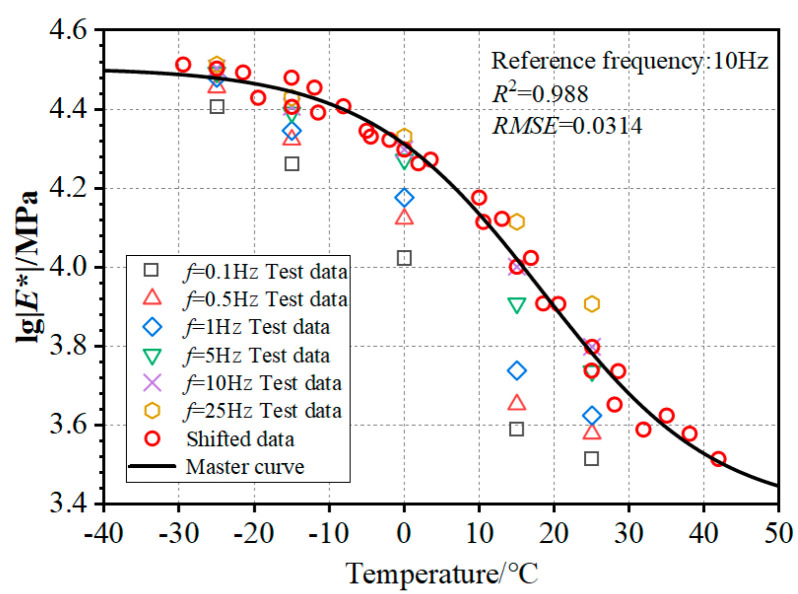
Dynamic modulus master curve at 10 Hz.

Table 6 and Table 7 outline the essential parameters for curve fitting related to the shift factor and dynamic modulus master curve, respectively. Figure 7 illustrates the dynamic modulus master curve at the fundamental frequency of 10 Hz. Equation (9) symbolizes the formulation of the dynamic modulus master curve.

(9) $$\mathrm{lg}|E^{*}|=3.258+\frac{1.173}{1+e^{(T-21.5)/10.08}}$$

For the viscoelastic material, the relaxation modulus *E*(*t*) is represented by the Prony series form of the generalized Maxwell model, as shown in Equation (10). Assuming the asphalt mixture is an isotropic material, *K*(*t*) and *G*(*t*) can be directly calculated from the relaxation modulus *E*(*t*) and Poisson’s ratio *ν* according to Equations (11) and (12).

(10) $$E(t)=E_{\infty }+\sum_{i=1}^{m}E_{i}e^{\frac{-t}{\rho_{i}}}$$

(11) $$K\left(t\right)=\frac{E\left(t\right)}{3\left(1-2v\right)}$$

(12) $$G\left(t\right)=\frac{E\left(t\right)}{2\left(1+v\right)}$$

where *E*(t)—relaxation modulus function; *E*_∞_—long-term relaxation modulus; *ρ_i_*—relaxation time; and *E_i_*—relaxation modulus corresponding to *ρ_i__._*

Obtaining the parameters *E*_∞_, *ρ_i_*, and *E_i_* for the Prony series is crucial for constructing the viscoelastic constitutive model of asphalt mixtures. In practical applications, Equation (10) is typically used to solve for the Prony series parameters of the relaxation modulus by applying the nonlinear least squares method to the frequency-domain dynamic modulus master curve of the asphalt mixture, as shown in Table 8.

#### 3.3.2. Fracture Energy

**Figure 8:**
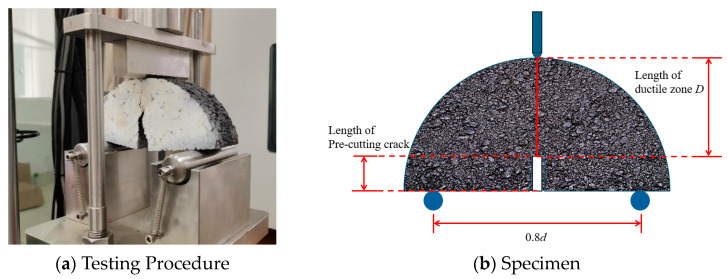
Semi-circular bending testing.

The fracture energy release rate *G_f_* constitutes a fundamental material property quantifying fracture resistance and damage evolution in asphalt mixtures [45]. Mechanical parameters such as *G_f_* and tensile strength (*S*) were obtained through semicircular bending (SCB) tests [27,46], with Figure 8 showing the experimental setup. The relationship between fracture work (*W_f_*) and *G_f_* is derived through Equation (13) [47,48,49]:

(13) $$G_{f}=\frac{W_{f}}{Dt}\times 10^{6}=\frac{\int Pdl}{Dl}\times 10^{6}$$

where *G_f_* means fracture energy release rate (J·m^−2^), *W_f_* is fracture work (J), *D* represents ductile zone length (mm), *t* serves as specimen thickness (mm), *P* means loads (kN), *l* is displacement (mm), and *d* is the diameter of the specimen.

This study employs the XFEM combined with a modified Paris’ law to investigate fatigue cracking of pavement asphalt layers. The critical energy release rate *G_f_* obtained from SCB tests was assigned as the fatigue parameter *G*_equivc_ [50]. This fracture parameter, along with the tensile strength *S*, was incorporated into the XFEM model as critical material inputs. Table 9 provides the XFEM parameters.

The thermo-mechanical coupling analysis incorporated the dynamic modulus master curve to characterize temperature-dependent variations in the asphalt surface layer, while material properties of other structural layers were considered temperature-invariant. Material parameters for all layers are systematically detailed in Table 10.

### 3.4. Validation

**Figure 9:**
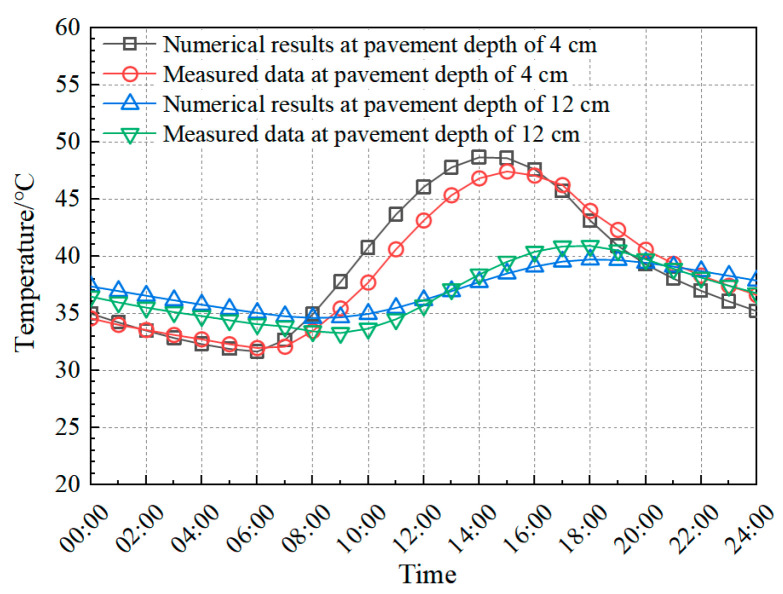
Comparison of field-measured temperature and FE model.

**Figure 10:**
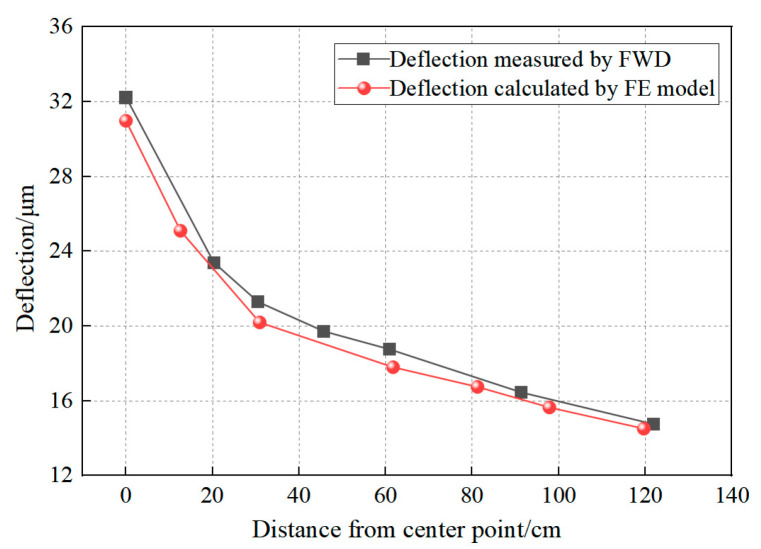
Comparison between the measured surface deflection of FWD and the surface deflection calculated by the FE model.

The FE model was validated through experimental temperature and deflection measurements. Temperature validation involved comparing simulated results with embedded sensor data from the RIOHTrack STR5 section, while mechanical validation contrasted FE-predicted deflections with field measurements obtained using the falling weight deflectometer (FWD). The temperature field validation results are presented in Figure 9, with deflection prediction accuracy demonstrated through FWD comparisons in Figure 10.

Comparative analysis of simulated and experimental thermo-mechanical parameters confirms the FE model predictive accuracy. Figure 9 demonstrates ≤3 °C maximum temperature deviation between numerical simulations and field measurements across pavement depth profiles. Figure 10 further validates the model through deflection basin comparisons, revealing <5% discrepancy between FE predictions and falling weight deflectometer (FWD) field data. Observed variations originate from three systematic sources: numerical solution algorithms in FE software (ABAQUS 2020), inherent material property heterogeneity, and transient environmental conditions (solar irradiance, wind velocity, humidity, and traffic loading patterns) [51,52,53]. These systematic variations remain within acceptable tolerance thresholds for pavement engineering applications. The validated FE model effectively simulates both temperature and stress fields in composite pavement under thermo-mechanical coupling.

## 4. Results and Discussion

### 4.1. Temperature–Modulus Gradient

#### 4.1.1. Temperature Gradient

**Figure 11:**
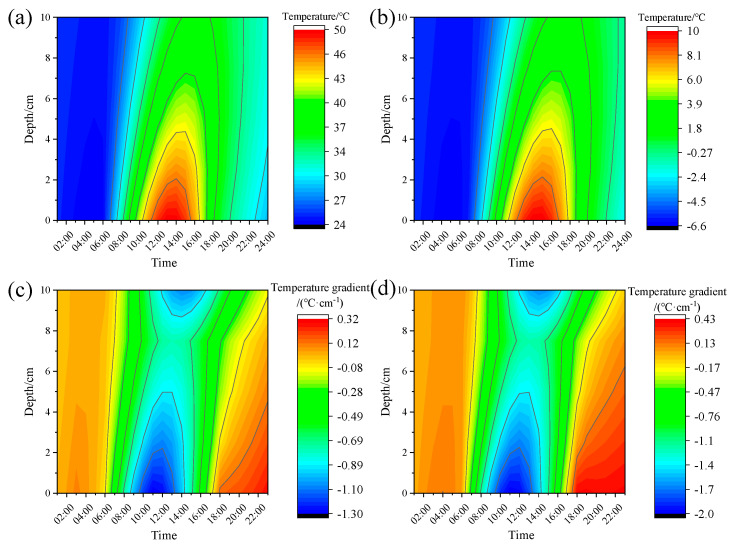
Non-uniform temperature field: (**a**) Summer asphalt layer temperature; (**b**) winter asphalt layer temperature; (**c**) summer asphalt layer temperature gradient; (**d**) winter asphalt layer temperature gradient.

Figure 11a,b show the daily temperature variation of the asphalt layer in composite pavement under summer and winter conditions, respectively, while Figure 11c,d depict the corresponding temperature gradient distributions. The thermal profile exhibits significant spatiotemporal variability across different depths. At 1:00 h, temperatures remain comparatively low (≈25 °C), showing minimal thermal stratification within the 0–10 cm depth range (ΔT < 0.5 °C·cm^−1^). At 08:00, solar loading induces pronounced heating in the upper asphalt layers, creating a negative thermal gradient due to thermal inertia in deeper sections. Maximum thermal differentials occur between 10:00 and 14:00, peaking at −1.96 °C·cm^−1^. The subsequent cooling phase (14:00–18:00) reduces this gradient to −0.47 °C·cm^−1^ through thermal equilibration, while maintaining elevated mean temperatures (≈36 °C) relative to nocturnal conditions.

The winter temporal evolution of internal temperature mirrors that of summer, yet with a smaller temperature and temperature gradient within the asphalt layer. At 1:00, 8:00, 10:00, 14:00, and 18:00, the average temperature gradients along the depth are 0.12 °C·cm^−1^, −0.44 °C·cm^−1^, −0.83 °C·cm^−1^, −0.87 °C·cm^−1^, and −0.11 °C·cm^−1^, respectively. Notably, the maximum temperature of the asphalt layer remains below 10 °C, within a lower temperature spectrum. Owing to the cold properties of asphalt mixtures, the linear elasticity assumption proves beneficial for simplifying the mechanical model in the stress analysis of composite pavement during winter.

The mechanical behavior of asphalt mixtures exhibits significant temperature dependence, leading to dynamic modulus stratification within pavement layers under thermal gradients [2]. Conventional mechanical characterization based on isothermal laboratory testing fails to account for this critical phenomenon, potentially introducing substantial discrepancies between design assumptions and in-service performance. Such oversimplification compromises both the analytical accuracy and engineering relevance of pavement structural evaluations.

#### 4.1.2. Dynamic Modulus Gradient

**Figure 12:**
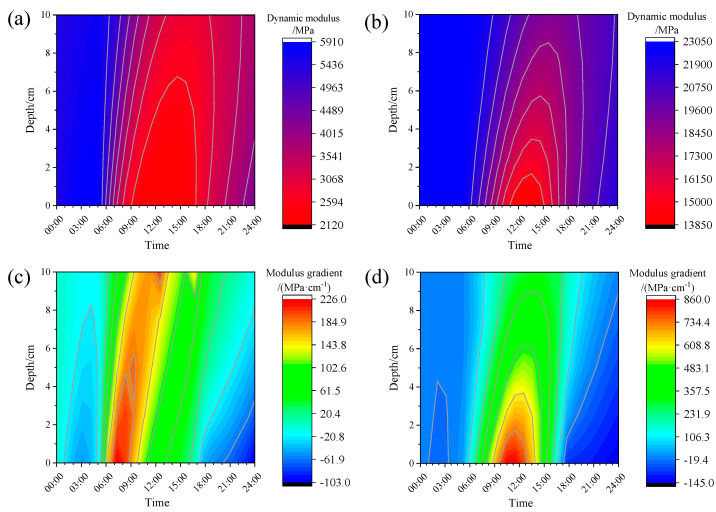
Non-uniform modulus field: (**a**) Summer asphalt layer modulus; (**b**) winter asphalt layer modulus; (**c**) summer asphalt layer modulus gradient; (**d**) winter asphalt layer modulus gradient.

Figure 12a,b demonstrate the spatiotemporal distribution characteristics of the asphalt layer’s dynamic modulus. Comparative analysis reveals a significant modulus enhancement during winter conditions relative to summer, while preserving consistent temporal variation patterns. The dynamic modulus exhibits strong thermal dependence, manifesting depth-dependent fluctuations that inversely correlate with temperature distribution. Surface regions (AC depth = 0 cm) display more pronounced modulus variations than deeper layers (AC depth = 10 cm), with elevated temperatures corresponding to reduced modulus values. These observations establish the foundation for deriving the dynamic modulus gradient distribution shown in Figure 12c,d.

A comparative analysis of spatiotemporal dynamic modulus gradient distributions reveals seasonally dependent characteristics. Although winter exhibits smaller thermal differentials within the asphalt layer than summer, the dynamic modulus gradient demonstrates greater depth-dependent variation during colder periods, attributable to reduced baseline temperatures. Quantitative measurements show maximum gradient values of 226 MPa·cm^−1^ (summer) versus 860 MPa·cm^−1^ (winter), with corresponding diurnal variation amplitudes of 329 MPa·cm^−1^ and 1005 MPa·cm^−1^, respectively.

The asphalt surface layer demonstrates the most pronounced thermal fluctuations, consequently exhibiting maximum modulus gradients at the surface. Temporally, significant dynamic modulus gradients in the asphalt mixture primarily manifest during diurnal heating periods (8:00–18:00). These observations underscore the necessity of incorporating time-dependent thermal effects in pavement mechanical analyses.

### 4.2. Thermal Stress

**Figure 13:**
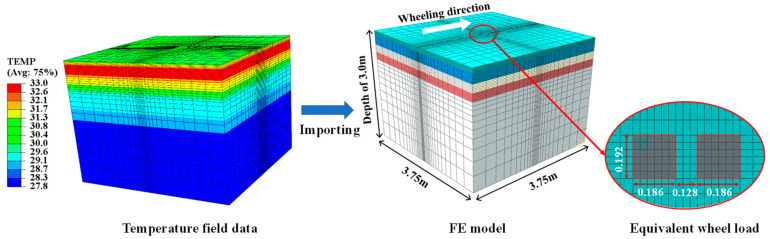
Schematic diagram of thermo-mechanical coupling process.

**Figure 14:**
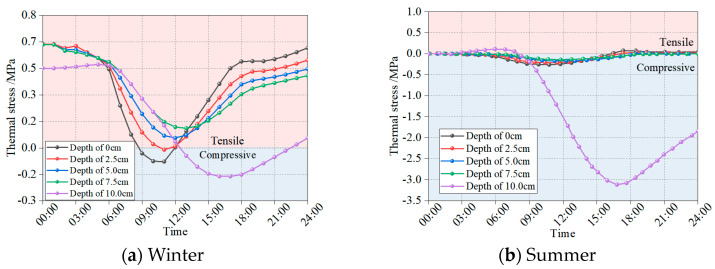
Thermal stress in asphalt layer of composite pavement.

The thermo-mechanical coupling model for the rigid–flexible composite pavement was developed using a sequential coupling methodology. The temperature field data, obtained from prior analysis, was incorporated as boundary conditions into the finite element analysis to simulate the thermal effects on the mechanical response. The applied load was the standard axle load BZZ-100, moving at a speed of 60 km/h. For simplification in the modeling process, the load was idealized as two rectangular footprints, each measuring 0.186 m by 0.192 m, with a spacing of 0.128 m between them. Figure 13 illustrates the sequential coupling procedure, while Figure 14 displays the temporal evolution of computed thermal stresses within the asphalt layer.

Figure 14 demonstrates that winter conditions induce significantly higher tensile stresses in the asphalt layer compared to the predominantly compressive stresses observed during summer. This seasonal variation stems from two key factors: (1) the enhanced dynamic modulus of asphalt mixtures at lower winter temperatures, and (2) thermally induced contraction (winter) versus expansion (summer) behavior. These observations establish winter as the critical period for evaluating pavement thermal stresses and investigating thermally induced cracking mechanisms. The analysis reveals particularly pronounced stress differentials at the flexible–rigid interface (h = 10 cm), attributable to the substantial modulus contrast between the asphalt and base layers. This mechanical discontinuity leads to incompatible deformations across the interface, which, under repeated thermal cycling, promotes fatigue cracking initiation in the asphalt layer. Such damage mechanisms may progressively develop into reflective cracking over prolonged service periods.

### 4.3. Load Stress

In contrast to winter conditions, the most significant dynamic modulus contrast between the asphalt layer and the rigid base layer occurs during summer, emphasizing the “flexible surface layer-rigid base layer” nature of the composite pavement. Therefore, this study primarily investigates the mechanical behavior of asphalt layers in composite pavement under summer temperature fields.

#### 4.3.1. Flexural–Tensile Stress

**Figure 15:**
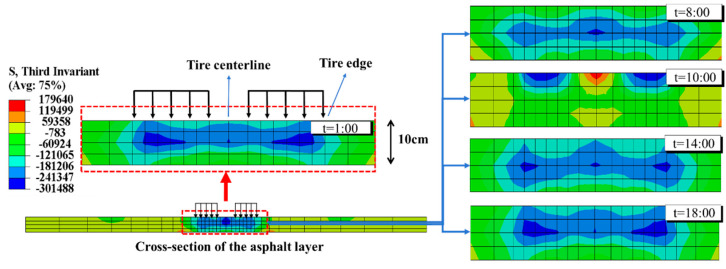
Third invariant of deviatoric stress J3 between 1:00 and 18:00.

The value of the third invariant of the deviatoric stress *J*_3_ determines the Lode angle *θ* on the π-plane within the principal stress space. When *J*_3_ > 0, tensile strain occurs in the element; when *J*_3_ = 0, plane strain occurs; and when *J*_3_ < 0, compressive strain occurs [54,55]. The distribution characteristics of *J_3_* are presented in Figure 15.

Figure 15 illustrates that, considering the transient temperature field at 1:00 in the early morning as an example, the *J*_3_ inside the asphalt layer in the load-affected region is negative. Additionally, the closer to the area of the dual-wheel load, the smaller the *J*_3_ of the asphalt layer. Temperature has a significant impact on the internal *J*_3_. Under transient temperature fields at different times, there are noticeable differences in the *J*_3_ response. However, the overall *J*_3_ inside the asphalt layer is predominantly negative. Combining the relationship between *J*_3_ and the stress state of the element, it can be inferred that under the combined action of load and temperature, the main deformation generated in the composite pavement is compressive. This finding aligns with the finite element analysis of asphalt layer strain in rigid–flexible composite pavements conducted by Ling [6], which similarly reported significant compressive strains generated beneath the two tires.

**Figure 16:**
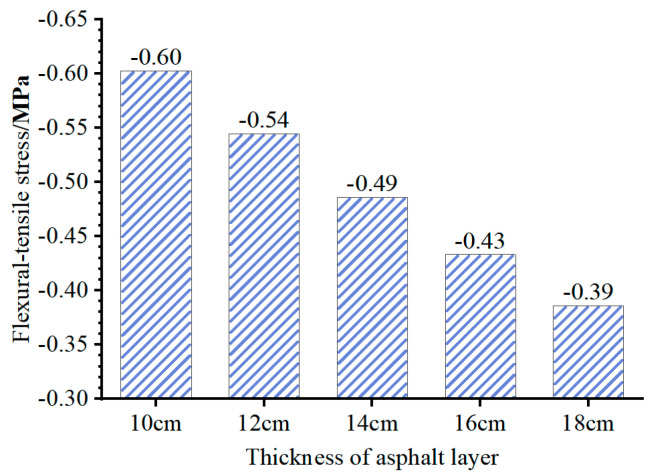
Flexural–tensile stress at the bottom of asphalt layer (*E*_base_ = 30,000 MPa).

**Figure 17:**
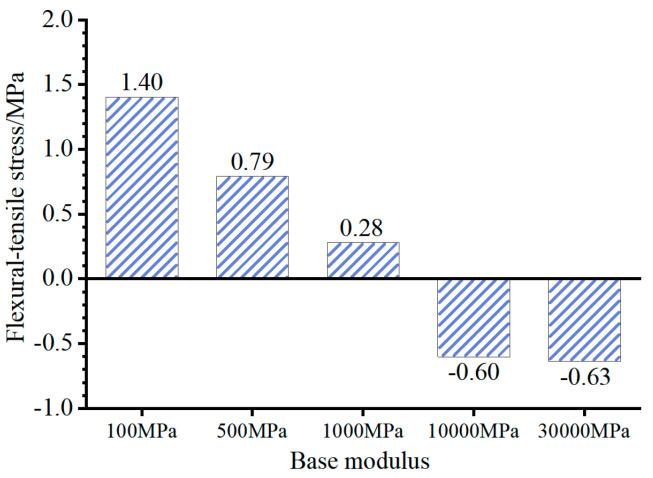
Flexural–tensile stress at the bottom of asphalt layer (*h*_AC_ = 10 cm).

The primary factor leading to distinct stress distributions between composite pavement and those with flexible bases is the structural amalgamation of a “high stiffness base + thin asphalt layer”. To investigate the impact of the base modulus (*E*_base_) and asphalt layer thickness (*h*_AC_) on the stress at the bottom of the asphalt layer, adjustments were made to the FE model to vary *E*_base_ and *h*_AC_. Subsequently, the flexural–tensile stress at the bottom of the asphalt layer under the summer temperature conditions at 14:00 was computed and is depicted in Figure 16 and Figure 17.

Figure 16 and Figure 17 illustrate that increasing the thickness of the asphalt layer can reduce the compressive stress at the layer’s bottom. However, for a base modulus *E*_base_ = 30,000 MPa, variations in asphalt layer thickness within the range of 10 to 18 cm do not impact the tensile or compressive state at the layer’s bottom. Notably, the base modulus significantly influences the tensile and compressive state at the bottom of the asphalt layer. With *E*_base_ of 10,000 MPa and 30,000 MPa (representative values for inorganic and cement-stabilized bases), the asphalt layer bottom experiences compression. In contrast, for *E*_base_ of 100 MPa, 500 MPa, or 1000 MPa (representative values for granular and asphalt-stabilized bases), the asphalt layer bottom undergoes tension. Consequently, in composite pavement featuring a thin asphalt layer and a high-stiffness base, utilizing the maximum tensile strain at the bottom of the asphalt layer as the design criterion does not align with the structure’s stress characteristics.

#### 4.3.2. Shear Stress

**Figure 18:**
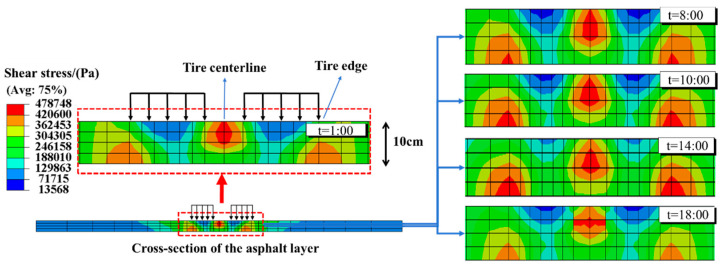
Shear stress of asphalt layer between 1:00 and 18:00.

Figure 18 shows the distribution of shear stress within the asphalt layer at different moments of the temperature field.

In the shear stress cloud at 1:00, the maximum shear stress within the upper asphalt layer occurs at the tire centerline, while the maximum shear stress at the bottom of the asphalt layer occurs at the tire edge. This distribution implies a susceptibility to top-down shear failure at the centerline and bottom-up shear failure near the tire edges. In Figure 18, across transient temperature fields from 8:00 to 18:00, the position of the maximum shear stress remains consistent. However, variations in asphalt material properties due to temperature fluctuations lead to changes in shear stress levels, with the lowest shear stress within the asphalt layer occurring at 14:00, the hottest period of the day.

**Figure 19:**
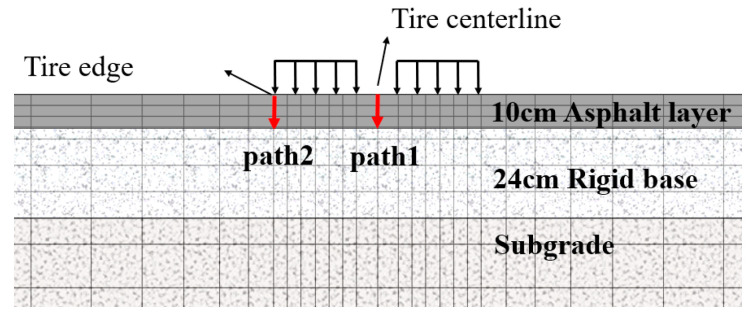
The schematic diagram of the internal shear stress observation path.

**Figure 20:**
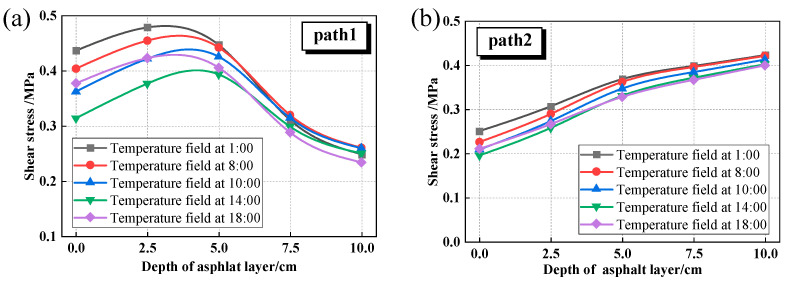
Shear stress under different temperature fields. (**a**) Path1; (**b**) Path2.

Figure 18 highlights the significance of the tire centerline and tire edge areas as critical areas for shear stress analysis. To assess the influence of temperature on the shear stress at these critical areas, two designated paths, Path 1 and Path 2, are delineated, as depicted in Figure 19. These paths facilitate the examination of shear stress variations within the asphalt layer across varying temperature fields. The shear stress analysis results are shown in Figure 20.

Figure 20 reveals distinct shear stress distributions between the tire centerline (Path 1) and the edge (Path 2) under diurnal temperature variations. The shear stress characteristics along Path 1 are consistent with those documented in the literature [56], showing an initial increase followed by a decrease with depth. In contrast, the shear stress along Path 2 gradually increases with depth. Path 1 exhibits a characteristic convex profile, with peak stress (0.479 MPa) occurring at 2.5 cm depth at 1:00 and decreasing to 0.393 MPa at 14:00. This 18% diurnal variation demonstrates significant thermal sensitivity in surface-proximal regions. In contrast, Path 2 shows depth-dependent stress accumulation, with maximum values stabilizing at 0.45 MPa at 10 cm depth across all temperature conditions, exhibiting less than 5% variation. Path 1’s shallow stress concentration results from combined thermal contraction and surface loading effects, while Path 2’s stable stress reflects the asphalt layer’s thermal insulation capacity. These findings demonstrate the critical depth dependence of thermal effects on pavement mechanical response.

**Figure 21:**
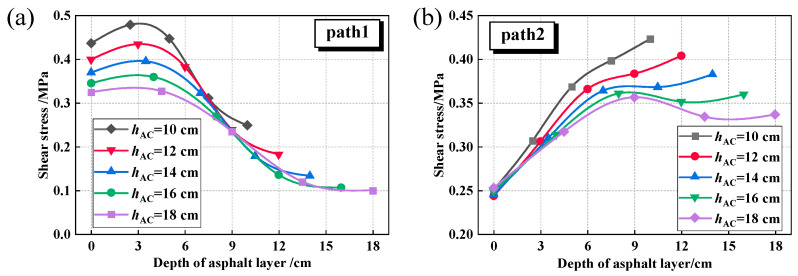
Shear stress under different asphalt layer thicknesses (*E*_base_ = 30,000 MPa). (**a**) Path1; (**b**) Path2.

The shear stress fluctuations within the asphalt layer of the composite pavement were also analyzed in this study for different *E*_base_ and *h*_AC_ conditions, as shown in Figure 21.

Figure 21a demonstrates that augmenting the *h*_AC_ leads to a notable reduction in shear stress levels. When the thickness increases from 10 cm to 18 cm, the maximum shear stress at the tire centerline decreases by 33.4%, while at the tire edge it decreases by 15.8%. Furthermore, Figure 21b illustrates that as the *h*_AC_ increases, the location of the maximum shear stress along Path 2 shifts from the bottom of the asphalt layer to the middle section.

**Figure 22:**
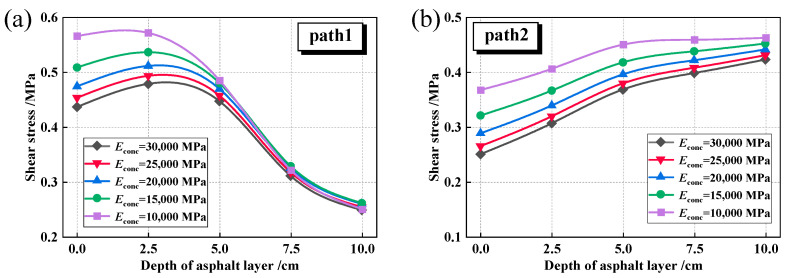
Shear stress under different base modulus (*h*_AC_ = 10 cm). (**a**) Path1; (**b**) Path2.

Figure 22 illustrates that with an increase in *E*_base_ from 10,000 MPa to 30,000 MPa, both the maximum shear stress at the tire centerline and at the tire edge exhibit a decreasing trend. Specifically, the maximum shear stress at the tire centerline decreases by 22.8%, while at the tire edge, it decreases by 8.6%. Consequently, compared to materials such as cement-stabilized macadam and asphalt-stabilized bases, utilizing higher modulus materials like cement concrete as the road base is more advantageous for thinner asphalt layers, effectively lowering the shear stress levels. This observation is consistent with the findings of Fan et al. [57], who also reported that the aging degree or modulus increase in the middle and lower asphalt layers contributes to reducing the overall shear stress level within the asphalt layer.

### 4.4. Cracking Analysis

This study utilized the XFEM to investigate the initial cracking behavior of the composite pavement asphalt layer subjected to thermal stress and wheel loading. Furthermore, the research included an examination of the cracking propagation characteristics of the asphalt layer under varying temperature field conditions.

#### 4.4.1. Cracking Characteristics

**Figure 23:**
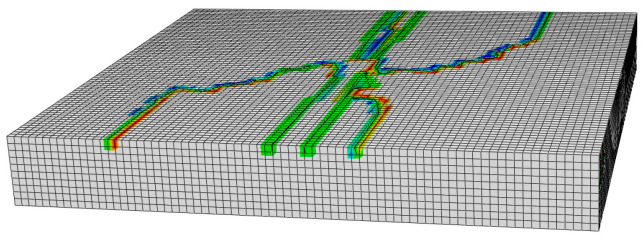
Three-dimensional view of crack initiation of asphalt layer.

**Figure 24:**
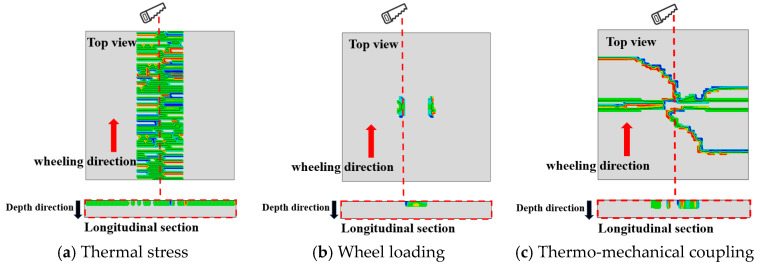
Initial cracking characteristics under different working conditions.

Figure 23 shows the three-dimensional picture of the crack initiation of the asphalt layer, and Figure 24 shows the cracking initiation characteristics under different working conditions. In order to make the picture description clearer, Figure 24 displays both the longitudinal section and top view of the asphalt layer along the wheel path direction, with the mesh removed for better visualization. The coloured areas indicate regions where cracks originate and propagate.

The comparison of asphalt layer cracking patterns under different working conditions, as depicted in Figure 24, reveals that thermal stress induces transverse surface cracking in the asphalt layer, while wheel loading results in longitudinal cracking along the tire periphery. Moreover, the asphalt layer, under the thermo-mechanical coupling, develops an intricate network of intersecting cracks, with diagonal cracking originating at the tire centerline and merging with transverse cracking at both ends. From the longitudinal section, it becomes apparent that cracking initiation primarily stems from the asphalt layer’s surface. This is due to the fact that the temperature fluctuations at the surface of the asphalt layer are the most dramatic in the actual service environment, which generates greater thermal stresses.

These observations are consistent with the field-measured top-down transverse cracking patterns in rigid-base asphalt pavements under thermo-mechanical coupling, as documented by [15]. Furthermore, the locations of crack initiation identified in this study align well with the field survey findings reported in [17]. Additionally, the cracking characteristics illustrated in Figure 24 exhibit more intricate details, highlighting the complexity of crack propagation under actual service conditions. These results collectively demonstrate that the proposed finite element modeling approach, which incorporates the coupling effect of temperature gradient and mechanical loading, proves capable of effectively simulating real-world cracking behavior in asphalt pavements.

**Figure 25:**
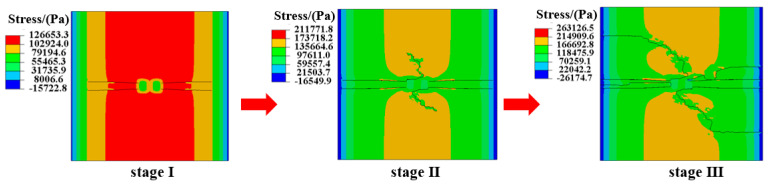
Crack propagation paths of surface under thermo-mechanical coupling.

In practice, the pavement structure is subjected to both thermal stress and wheel load stress. Therefore, the development characteristics of crack initiation and propagation on the surface of the asphalt layer under thermo-mechanical coupling were investigated, as depicted in Figure 25.

Figure 25 illustrates the impact of thermo-mechanical coupling on crack development at the asphalt layer surface. Initially, transverse cracks (stage I) emerge, followed by the gradual appearance of longitudinal cracks aligned with the wheel loading direction (stage II). Subsequently, these longitudinal cracks intersect with the transverse cracks, forming a network of interconnected cracks (stage III). Thermal stress predominantly drives crack initiation, while loading influences the crack propagation direction on the asphalt layer surface.

**Figure 26:**
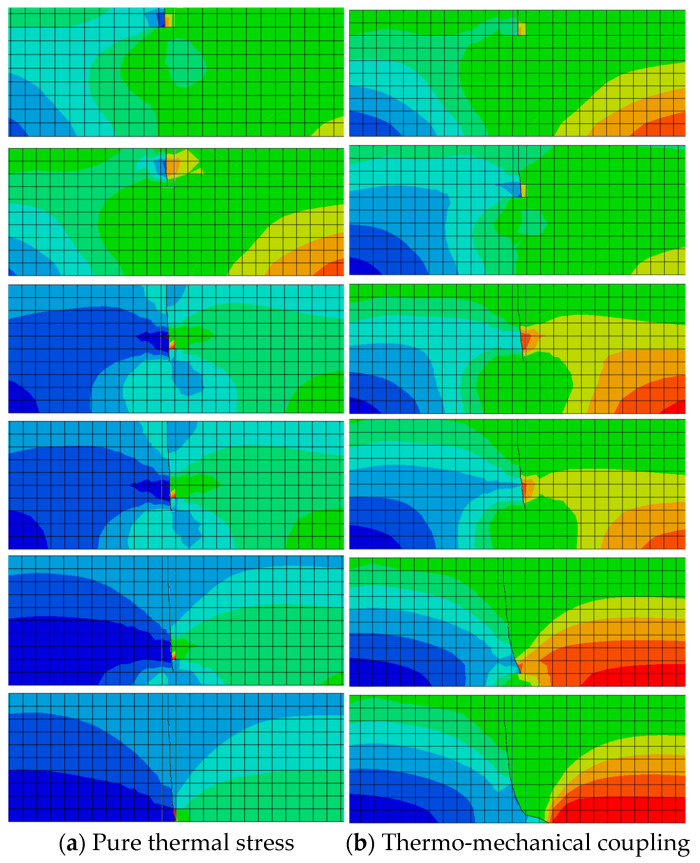
Top-down cracking propagation paths under different working conditions.

To investigate crack propagation patterns after initiation through the depth of the asphalt layer, a one-centimeter-deep transverse crack was created at the surface. The fatigue propagation pattern of this initial crack in the depth direction was analyzed, as illustrated in Figure 26.

Figure 26 demonstrates that following crack initiation, propagation continues under thermal stress or thermo-mechanical coupling until complete penetration of the asphalt layer occurs, forming top-down cracking. Under pure thermal stress, top-down cracking exhibits downward extension from the surface in Mode I (opening mode), ultimately penetrating the full layer thickness, with crack propagation direction perpendicular to the thermal stress. Due to asymmetric pavement loading, thermo-mechanical coupling induces mixed Mode I-II cracking, showing downward propagation along the tire edge at an inclined angle.

**Figure 27:**
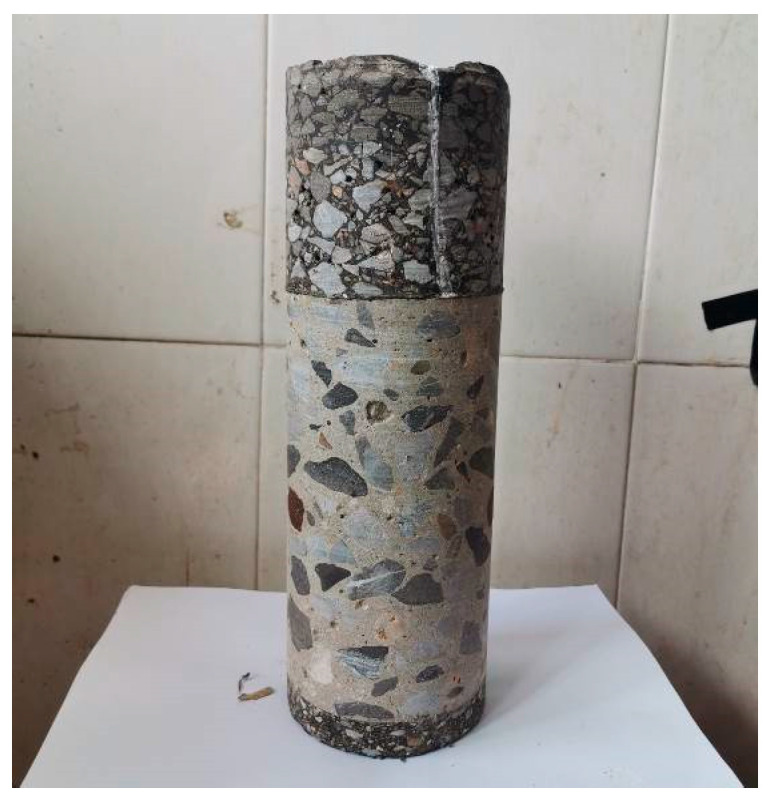
Top-down cracking core sample of Chang-Tan Expressway.

Subsequently, this study conducted observations and core sampling on the composite pavement of the Chang-Tan Expressway in Hunan Province, as depicted in Figure 27. The observed top-down cracking propagation path deviates from the vertical direction, exhibiting a certain degree of inclination, which is consistent with the expansion path simulated by finite element analysis.

#### 4.4.2. Fatigue Life

Hassan’s study [27] highlighted that the stiffness of the surface layer has the most significant influence on top-down fatigue life. Variations in the temperature field concurrently affect both the stiffness and fracture toughness of asphalt materials. This finding provides an important theoretical basis for the results of the present study. Building upon this, the present study further analyzed the effects of thermal stress and temperature fields at different time points on crack propagation characteristics. The thermal stress was incorporated into the XFEM model as a body stress. The fracture energy release rate *G_f_* at the crack tip and the number of load cycles required for complete crack penetration through the asphalt layer were selected as evaluation criteria.

(1) Effect of temperature field

**Figure 28:**
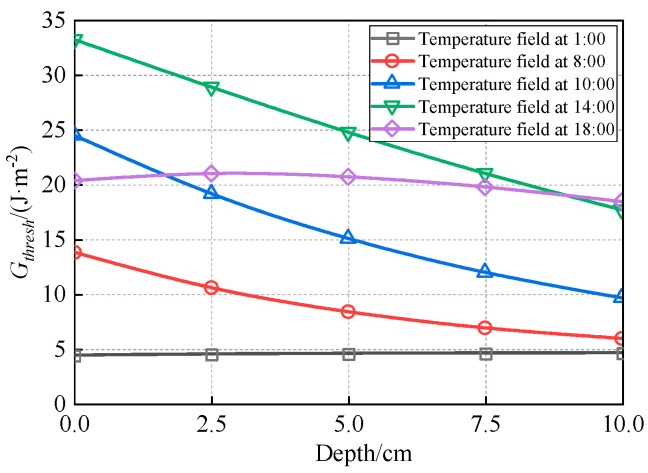
Distribution of *G_thresh_* along depth.

**Figure 29:**
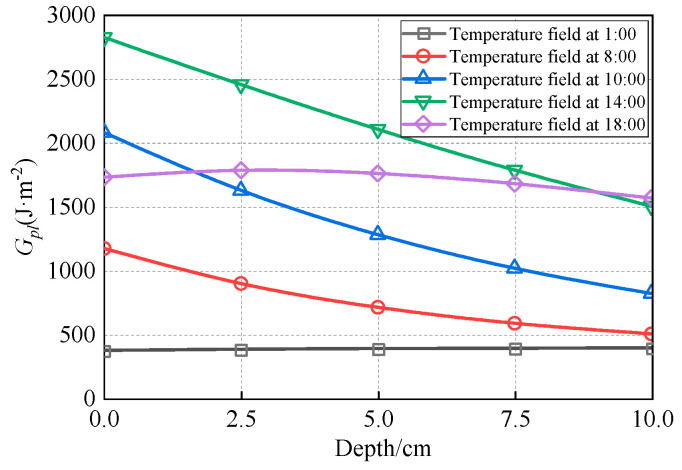
Distribution of *G_pl_* along depth.

**Figure 30:**
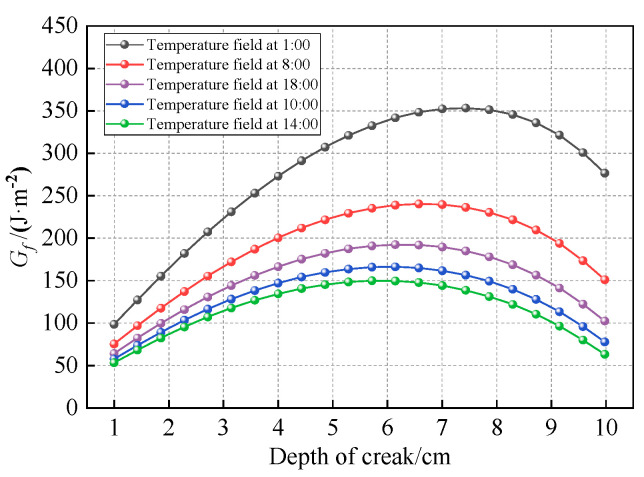
The *G_f_* under different temperature fields.

**Figure 31:**
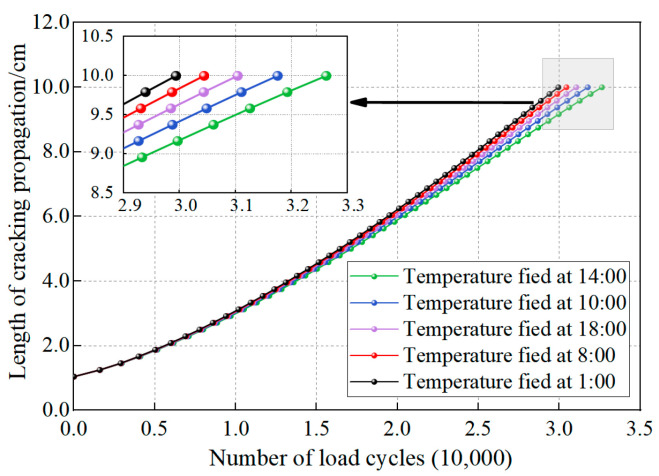
The number of load cycles under different temperature fields.

Using the winter temperature field depicted in Figure 11 and the fracture energy parameters from Table 9, the through-thickness distributions of *G_thresh_* and *G_pl_* within the asphalt layer were calculated. These distributions are visually represented in Figure 28 and Figure 29, respectively. The fracture energy release rate *G_f_* is illustrated in Figure 30, while Figure 31 presents the number of load cycles required for complete crack penetration.

Figure 30 demonstrates that the initial fracture energy release rate at the crack tip under varying temperature conditions ranges between 50 and 100 J·m^−2^. These values exceed the corresponding *G_thresh_*, causing top-down cracking to progress directly from the 1 cm pre-crack length into the medium-velocity stable propagation phase (Stage II of the modified Paris’ law curve).

When the crack propagates to approximately 6–7.5 cm, the fracture energy release rate *G_f_* reaches its maximum value of 350 J·m^−2^ under the 1:00 temperature condition. However, this value remains below the critical energy release rate (*G_pl_* = 390 J·m^−2^) required for fatigue crack progression into the unstable propagation stage. Conversely, the minimum *G_f_* of 155 J·m^−2^ occurs under the 14:00 temperature condition, which is significantly lower than its corresponding *G_pl_* (1700 J·m^−2^). Under none of the investigated temperature conditions did *G_f_* values reach their respective *G_pl_* thresholds, resulting in top-down cracks primarily propagating in Stage II (medium-velocity stable growth phase).

Figure 31 reveals a strong correlation between the fracture energy release rate *G_f_* and the number of load cycles required for complete crack penetration through the asphalt layer. The data demonstrate an inverse relationship where lower energy release rates correspond to extended structural fatigue life, confirming the effectiveness of the modified Paris’ law using *G_f_* in controlling top-down crack propagation. Analysis of the cracking process shows that the minimum *G_f_* value occurs under the 14:00 temperature condition, corresponding to the maximum fatigue life of 32,625 cycles. Conversely, the maximum *G_f_* appears under the 1:00 temperature condition, resulting in the minimum fatigue life of 29,933 cycles. These findings clearly indicate that temperature conditions significantly influence the crack-tip *G_f_*, which in turn affects crack fatigue life, with the observed 9.0% variation in fatigue life being directly attributable to temperature differences. Compared to the methodologies proposed in references [35,57], this study provides a novel contribution by incorporating the influence of non-uniform temperature fields into crack propagation analysis, rather than assuming a uniform temperature distribution.

(2) Effect of thermal stress

**Figure 32:**
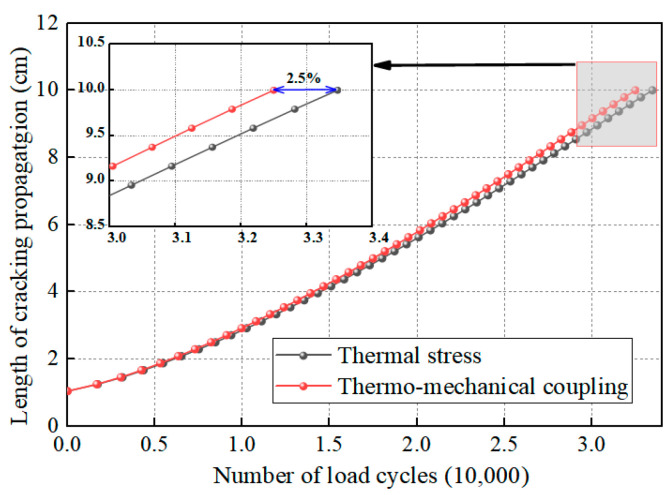
Fatigue life under different working conditions.

Figure 32 illustrates the relationship between crack propagation length and load cycles under thermo-mechanical coupling, compared with propagation under thermal stress alone. The results show that the fatigue life decreases from 33,472 cycles under pure thermal stress to 32,625 cycles when the crack propagates from 1 cm to 10 cm under thermo-mechanical coupling, representing only a 2.5% reduction. This minimal difference demonstrates that wheel loading primarily alters the fatigue propagation mode of top-down cracking from Mode I (opening mode) to mixed Mode I-II cracking, rather than significantly affecting the fatigue life.

## 5. Conclusions

This study developed a thermo-mechanical coupling analysis model for rigid–flexible composite pavements, incorporating temperature–modulus gradient differentials induced by environmental thermal variations. The model analyzes combined compressive-shear stress behavior in asphalt layers under thermal gradients and investigates cracking mechanisms through XFEM simulations coupled with a modified Paris’ law formulation. Key findings include the following:

(1) The asphalt layer exhibits significant modulus gradients induced by thermal gradients of −1.96 °C·cm^−1^ (summer) and −0.87 °C·cm^−1^ (winter). The daily modulus variations reach 329 MPa·cm^−1^ during summer and 1005 MPa·cm^−1^ in winter, with most pronounced fluctuations occurring at the surface. Using constant-temperature material parameters for pavement analysis and design leads to substantial deviations from actual service conditions, compromising analytical accuracy.
(2) Under thermo-mechanical coupling conditions, the asphalt layer develops predominantly compressive-shear stress states. The analysis reveals critical shear stress concentrations at both tire centerlines and edges, with nighttime (01:00) surface stress magnitudes being 18% higher than corresponding daytime (14:00) values. The stress distribution shows significant dependence on base layer stiffness. Increasing the modulus from 10,000 MPa to 30,000 MPa results in maximum shear stress reductions of 22.8% at centerline locations and 8.6% at edge positions.
(3) Asphalt layer cracking initiation predominantly occurs at the surface, exhibiting three distinct failure modes: (a) transverse cracking from thermal stress, (b) longitudinal cracking along traffic direction under loading, and (c) mixed-mode I–II fracture under thermo-mechanical coupling. Thermal stress governs crack initiation, while load stress controls propagation paths. Field validation via core sampling confirmed the inclined top-down cracking paths under coupled thermo-mechanical conditions.
(4) The fracture energy release rate G*_f_* at the crack tip exhibits significant temperature dependence. Under the 14:00 temperature field, *G_f_* reaches its minimum value (155 J·m^−2^), corresponding to the maximum fatigue life of 32,625 cycles. Conversely, at 01:00 conditions, *G_f_* attains its peak value (350 J·m^−2^), resulting in a reduced fatigue life of 29,933 cycles. This temperature-induced variation accounts for a 9.0% difference in fatigue performance.

Based on the findings of this study, future research in both scientific and applied engineering should focus on developing more sophisticated constitutive models and failure criteria to accurately characterize the crack propagation behavior of asphalt mixtures under the coupled influence of complex temperature fields and dynamic loading, given the significant temperature dependence of fracture toughness and modulus parameters. Concurrently, paramount emphasis should be placed on pioneering smart responsive pavement material systems—such as self-temperature-regulating and self-healing asphalt materials—to substantially enhance the adaptive capacity and long-term durability of pavement infrastructures under extreme climate conditions.

## Figures and Tables

**Table 1:** Tested 24 h temperature data.

Summer				Winter			
Time	Temperature/°C	Time	Temperature/°C	Time	Temperature/°C	Time	Temperature/°C
1:00	31.3	13:00	35.2	1:00	−2.5	13:00	3.6
2:00	31.0	14:00	35.6	2:00	−3.6	14:00	4.6
3:00	30.5	15:00	36.3	3:00	−5.1	15:00	5.4
4:00	30.3	16:00	37.2	4:00	−6.5	16:00	5.6
5:00	30.1	17:00	36.6	5:00	−7.1	17:00	5.3
6:00	28.4	18:00	36.6	6:00	−3.7	18:00	4.2
7:00	28.3	19:00	34.9	7:00	−2.3	19:00	3.2
8:00	30.4	20:00	33.7	8:00	−1.9	20:00	2.0
9:00	32.0	21:00	33.4	9:00	−1.0	21:00	1.7
10:00	31.4	22:00	32.4	10:00	0.4	22:00	1.2
11:00	32.5	23:00	32.0	11:00	2.1	23:00	0.7
12:00	33.7	24:00	31.0	12:00	2.8	24:00	0.2

**Table 2:** Meteorological parameters.

Month	Daily Solar Radiation Q/(J·m^−2^)	Sunshine Duration *c*/h	Daily Average Wind Speed *v*/(m·s^−1^)
December	9.14 × 10^6^	6.5	2.9
July	18.7 × 10^6^	11.7	2.5

**Table 3:** Thermodynamic parameters of materials [39].

Properties	AC	Cement Concrete	CSM	Soil
Thermal conductivity *k* (J/m·h·°C)	4670	6200	5620	5620
Density *ρ* (kg·m^−3^)	2300	2400	2200	1800
Thermal capacity *C* (J/kg·°C)	924	780	911	1040
Absorption factor of solar radiation *a*_s_ (%)	0.9			
Road surface emissivity *ε* (%)	0.81			
Absolute zero value (°C)	−273			
Stefan–Boltzmann constant (J/h·m^2^·K^4^)	2.041 × 10^−4^			

Note: CSM stands for Cement-Stabilized Material, e.g., cement-stabilized graded crushed stone and cement-stabilized soil.

**Table 4:** Testing program.

Testing Temperature/°C	Testing Frequency/Hz	Strain Level/με	Number of Parallel Tests
−25, −15, 0, 15, 25	0.1, 0.5, 1,5, 10, 25	85–95	4

**Table 5:** Dynamic modulus testing results.

Temperature/°C	Dynamic Modules/MPa					
	25 Hz	10 Hz	5 Hz	1 Hz	0.5 Hz	0.1 Hz
−25	32,359	31,623	30,903	30,200	28,184	25,704
−15	26,915	25,704	24,547	22,387	20,893	18,197
0	21,380	19,953	18,621	15,136	13,183	10,471
15	12,882	10,000	8128	5495	4467	3890
25	8128	6310	5495	4169	3802	3236

**Table 6:** Shift factor of dynamic modulus master curve.

Frequency/Hz	25	10	5	1	0.5	0.1
Shift Factor lg(*a_f_*)	−4.4926	0	3.4950	9.9493	12.3931	16.8551

**Table 7:** Parameters of dynamic modulus master curve.

lg|E^*^_max_|	lg|E^*^_min_|	*T*_E0_	*d*_x_
4.431	3.258	21.5	10.08

**Table 8:** Prony series parameters of relaxation modulus.

ρ_i_	Time/s	E_i_	Modulus/MPa
*ρ*_1_	0.000001	*E*_1_	5538
*ρ*_2_	0.00001	*E*_2_	6188
*ρ*_3_	0.0001	*E*_3_	6475
*ρ*_4_	0.001	*E*_4_	2712
*ρ*_5_	0.01	*E*_5_	1216
*ρ*_6_	0.1	*E*_6_	713
*ρ*_7_	1	*E*_7_	404.3
	∞	*E*_∞_	391.4

**Table 9:** Parameters of XFEM.

Temperature/°C	Tensile Strength *S*/MPa	Critical Energy Release Rate *G*_equivc_/(J·m^−2^)
5	5.16	2246
0	10.38	1792
−5	11.92	452

**Table 10:** Mechanical parameters [39].

Materials	Density/(kg·m^−3^)	Young’s Modulus/MPa	Poisson’s Ratio
Asphalt concrete	2400	Using dynamic modulus master curve	0.20
Portland cement concrete	2300	30,000	0.15
Cement-stabilized graded crushed stone	2300	1200	0.25
Cement-stabilized soil	2200	1000	0.30
Subgrade	1800	60	0.40

## Data Availability

The original contributions presented in this study are included in the article. Further inquiries can be directed to the corresponding author.

## Glossary

XFEM	extended finite element method
RIOHTrack	full-scale pavement test track
STR5	rigid base asphalt pavement structure in RIOHTrack
FWD	falling weight deflectometer
*G*	energy release rate
*K*	stress intensity factor
$C^{\prime }$	material parameters in the modified Paris’ law
$m^{\prime }$	material parameters in the modified Paris’ law
*G_thresh_*	threshold value of the fracture energy release rate
*G_pl_*	upper limit of the fracture energy release rate
*G_equivc_*	critical energy release rate
*G_f_*	fracture energy release rate
*W_f_*	fracture work
SPT	simple performance tester
*J*_3_	the third invariant of the deviatoric stress
*E*_base_	modulus of base
*h*_AC_	thickness of asphalt layer
SCB	semicircular bending tests
